# Using airbrushes to pattern reagents for microarrays and paper-fluidic devices

**DOI:** 10.1038/micronano.2017.55

**Published:** 2017-11-20

**Authors:** Christopher L. Cassano, Teodor Z. Georgiev, Z Hugh Fan

**Affiliations:** 1 Interdisciplinary Microsystems Group, Department of Mechanical and Aerospace Engineering, University of Florida, P.O. Box 116250, Gainesville, FL 32611, USA; 2 J. Crayton Pruitt Family Department of Biomedical Engineering, University of Florida, P.O. Box 116131, Gainesville, Florida 32611, USA; 3 Department of Chemistry, University of Florida, P.O. Box 117200, Gainesville, FL 32611, USA

**Keywords:** airbrush, paper-fluidics, fabrication, microarrays, lateral flow assays, chemiluminescence

## Abstract

We report using an airbrush to pattern a number of reagents, including small molecules, proteins, DNA, and conductive microparticles, onto a variety of mechanical substrates such as paper and glass. Airbrushing is more economical and easier to perform than many other patterning methods available (for example, inkjet printing). In this work, we investigated the controllable parameters that affect patterned line width and studied their mechanisms of action, and we provide examples of possible patterns. This airbrushing approach allowed us to pattern lines and dot arrays from hundreds of μm to tens of mm with length scales comparable to those of other patterning methods. Two applications, enzymatic assays and DNA hybridization, were chosen to demonstrate the compatibility of the method with biomolecules. This airbrushing method holds promise in making paper-based platforms less expensive and more accessible.

## Introduction

The high cost of medical diagnostics and the importance of detecting infectious diseases in a timely, cost-effective manner have increased the importance of point-of-care (POC) testing. Lateral flow assays, including the commonly used pregnancy test, are among the best examples of effective POC testing, because they are simple, inexpensive, and have minimal training requirements^1,2^. The extension of lateral flow assays into paper-based microfluidic devices in the past decade has created new research interests^2–4^. DNA and protein microarrays are very powerful and have the potential to become useful methods for detection and diagnosis of disease in POC settings^5–7^. In all of these cases, small volumes of reagents must be deposited onto a mechanical substrate such as paper, glass (for example, microscope slide), or plastic in a consistent, uniform, and reproducible manner.

In a commercial context, purpose-built piezoelectric printers or similar items are used to accomplish the task of dispensing reagents. They have high resolution and they are fast, accurate, and reproducible. However, they are too expensive to use in a typical research lab. Commercially available microarray instruments have a price ranging from $30* *000 to $100* *000^8^, and thus they are generally limited to specialized laboratories.

To address the need for low-cost equipment for fabricating paper-based microfluidic devices, several research groups have developed creative ways to deposit reagents on mechanical substrates, including plotter pens^9^, syringe tips^10^, contact printers (for example, dot matrix printers and split nib pens)^11–13^, screen printing^14,15^, and bubble jet and inkjet printers^16–18^. Although all of these methods are useful, they all have some drawbacks.

For example, contact-based methods including plotter pens, syringe tips, and some types of contact printers (for example, split nib designs) can cause physical damage to the surface during reagent deposition, especially on paper. In addition, capillary forces through paper’s porous structures drive the flow of fluid from the printer’s contact location through the substrate. As a result, the quality of the printing is strongly dependent on the porosity and hydrophobicity of the substrate.

Non-contact printers, including bubble jets and inkjets, avoid the pitfalls of the contact printing methods; however, consumer-grade printers^19,20^ consume a large amount of reagents (typically hundreds of microliters) during priming and cleaning operations. We attempted this method before conducting this work to verify this limitation. For the average laboratory environment in which only a few devices are made at a time, the waste resulting from priming could exceed the required reagent volume (typically a few microliters) by 20 to 30 times, or more. Inkjet print heads cannot be run dry without risking damage; hence, the internal reservoir must remain full at all times. Because it is very difficult to recover unused reagents from the internal reservoir, the total volume of wasted reagents is even higher. Given the cost of reagents (such as antibodies used in lateral flow assays), this type of printing can become prohibitively expensive.

As a result, we investigated the use of airbrushes to deposit reagents on mechanical substrates. An airbrush is an art instrument that is often used in tattoo shops for creating patterns on human bodies^21^; only a small amount of ink is needed. Airbrushes have been scientifically used for cocaine delivery to fruit flies^22^, fabricating microelectrodes for electrochemical detection^23^, insecticide applications to crop leaves^24^, layer-by-layer growth of perovskite thin films^25^, and spraying carbon nanotubes^26^. An airbrush typically costs approximately $100, which is 2 to 3 orders of magnitude less expensive than microarray spotters.

In this report, we describe our efforts in patterning a number of reagents, including small molecules, proteins, DNA, and conductive microparticles, onto a variety of mechanical substrates such as paper and glass. Lines, dot arrays, letters, and artistic patterns have been created by airbrushing. We also report our investigation in the controllable parameters that affect the patterned line width. In addition, we demonstrated the applications of enzymatic assays and DNA hybridization for flu virus detection with reagents airbrushed on paper substrates.

## Materials and methods

### Materials and reagents

Whatman CHR1 chromatography paper in 2* *cm wide roll format was obtained from Fisher Scientific (Pittsburgh, PA, USA). Glutaraldehyde 50% solution, 10× phosphate*-*buffered saline, sodium hydroxide, Tween-20, tris(hydroxymethyl)aminomethane (TRIS), sodium citrate, horseradish peroxidase (HRP), sodium dodecyl sulfate, and sodium citrate dihydrate were also obtained from Fisher Scientific. Low*-*molecular*-*weight chitosan (>75% deacetylation), carboxymethyl cellulose (CMC), bovine serum albumin (BSA), CDP-Star substrate solution, and glycine were obtained from Sigma-Aldrich (St Louis, MO, USA). Reagent-grade sodium chloride, magnesium chloride, ethylenediaminetetraacetic acid (EDTA), luminol, and ammonium chloride were obtained from Acros Organics (Morris Plains, NJ, USA). Red food dye was obtained from McCormick & Company, Inc. (Sparks, MD, USA). SGC8 aptamer^27^ (6FAM-5′- ATCTAACTGCTGCGCCGCCGGGAAAATACTGTACGGTTAGATTTTTTTTTT-3′-NH_2_) was synthesized in house. Oligonucleotides were purchased from Integrated DNA Technologies (Coralville, IA, USA). Triton-X100 and alkaline phosphatase-streptavidin conjugate (RPN1234) were obtained from Amersham Biosciences (Piscataway, NJ, USA). Tetramethylbenzidine (TMB) membrane peroxidase substrate was obtained from KPL Inc. (Gaithersburg, MD, USA). Hiperco 50 (6* *μm particles from an alloy containing cobalt, vanadium, and iron) was obtained from Carpenter Technology (Wyomissing, PA, USA). Carbon ink paste (BQ225) was obtained from DuPont (Research Triangle Park, NC, USA). Solutions were prepared using deionized water obtained from a Barnstead Thermolyne Nanopure Diamond ultraviolet/ultra-filter water purification system (Asheville, NC, USA).

For creating lines and dot arrays on paper or glass, a solution containing 0.1 wt-% (weight percentage) Triton X-100 and 0.1 wt-% CMC was made and combined with a red food dye solution (33 volume-%) or HRP (0.25* *mg mL^−1^). An aliquot of 250* *μL of this solution was pipetted into the reservoir of the airbrush.

For using conductive carbon ink, the ink was first diluted with acetone to obtain a final concentration of 0.1* *g* *mL^−1^. For Hiperco 50 powder, the ink was prepared in a solution containing 1* *g mL^−1^ microparticles in a solution of 0.1 wt-% Triton X-100 and 0.1875 wt-% CMC. Each solution was vortexed for 30* *s, and an aliquot of 250* *μL was pipetted into the reservoir of the airbrush.

### Airbrush and its operation

**Figure 1:**
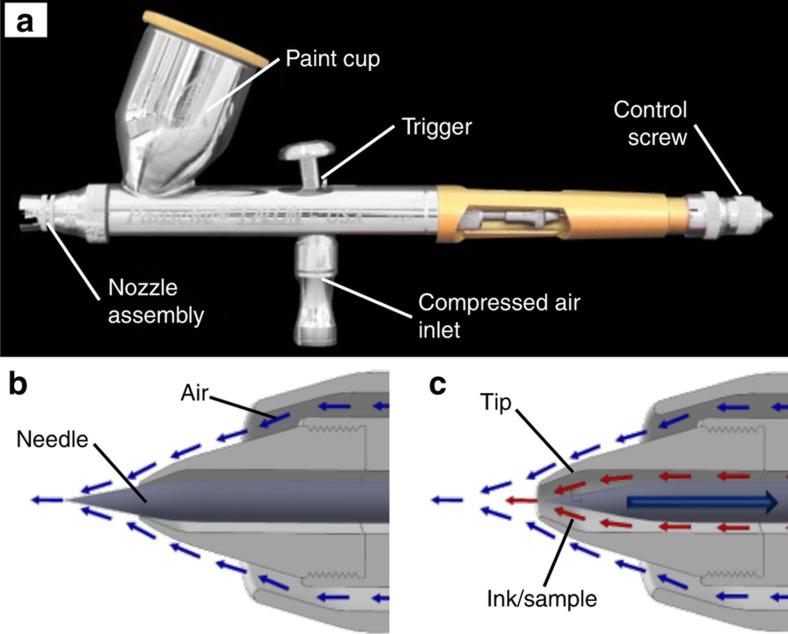
(**a**) Picture of an airbrush with key components marked. It consists of a paint cup for reagents, a nozzle assembly for spraying, a trigger to turn on compressed air, and a control screw to adjust the nozzle. (**b**) When the airbrush needle valve is closed, air exits around the tip as indicated by the blue arrows but no liquid can leave the nozzle. (**c**) After the needle valve is opened by withdrawing towards the left via the control screw, the air rushing past the tip entrains liquid (red arrows) contained in the reservoir, and a fine spray is created.

All patterning was conducted using a Paasche Talon airbrush (Paasche Airbrush Company, Chicago, IL, USA) fitted with a TT-1 (0.25* *mm) airbrush tip and TN-1 (0.25* *mm) needle. The picture of the airbrush is shown in Figure 1a. The paint cup could be much smaller, as in another model of airbrush that we have previously described^23^. After the paint cup is filled with a reagent or ink, a fine mist of atomized liquid is created by the Venturi effect through the stream of the compressed air, which starts to flow by pressing the trigger. We used compressed air from the building, although a gas tank or an air compressor may be used.

As shown in Figures 1b and c, the airbrush has two concentric annular nozzles: an outer nozzle through which air passes and an inner nozzle through which solution passes. A tapered needle slides in and out of the inner nozzle via a control screw, acting as a valve. When the tapered needle valve moves back, a gap forms between the needle and the nozzle orifice, and the farther back the needle valve moves, the larger the orifice becomes. During operation, the shape of the outer nozzle causes a partial vacuum to develop at the interface between the moving stream of air and the tip. Liquid is drawn from the reservoir, owing to the pressure difference between the ink and the air stream.

The simplest way to make patterns by using an airbrush is to draw them by hand as is done in tattoo shops. Drawing by hand is easy to do, but the quality of the patterns produced depends upon the operator’s skill. To improve the reproducibility of patterns produced using an airbrush, an automated translation system was assembled. For one-dimensional translation, we fixed the substrate onto the pusher block of a syringe pump while the airbrush was fastened to a stand. For complex patterns, we used a desk top CNC machine to achieve either two- or three-dimensional translations.

Except for the conditions specified otherwise, the airbrush was operated with an air pressure set at 50* *psi. The needle valve was adjusted by closing it fully and then drawing it back until the needle valve just began to release the fluid. The airbrush was programmed to traverse a predefined path at 30* *mm min^−1^ for lines or 100* *mm min^−1^ for dots.

### Protein assays and DNA hybridization

After HRP was airbrushed on a paper substrate, the solution was allowed to dry completely in the ambient environment for ~45 min. For colorimetric detection, the paper was placed in a petri dish, covered in 500* *μL of the TMB indicator solution, and allowed to soak for 10 min. The excess indicator was removed and the strips were rinsed in 2* *mL of deionized water. After being rinsed, the strips were dried and photographed using a digital SLR camera (EOS Rebel T3i, Canon USA, Melville, NY, USA).

For chemiluminescence detection, the paper with dried protein patterns was placed in a petri dish and covered in 500* *μL of freshly made luminescence solution (11.25* *mM luminol and 1.75% hydrogen peroxide). The paper was then placed inside a dark box and a 30-s exposure was taken using the camera.

For DNA hybridization, paper strips were prepared for immobilization of DNA by using a strategy presented elsewhere for the immobilization of protein on cellulose paper^28,29^. In brief, paper strips measuring 40* *mm long and 10* *mm wide were covered in 30* *μL of 0.25* *mg mL^−1^ chitosan and allowed to dry at room temperature. The paper strips were then covered in 50* *μL of 2.5% glutaraldehyde and allowed to soak at room temperature. After being rinsed with de-ionized water, the paper strips were allowed to dry at room temperature before DNA was applied.

Amine-terminated DNA was dialyzed in pure water, then diluted to obtain a working concentration of 5* *μM. The DNA was then mixed with a bioink base^30^ containing CMC and Triton-X100 to obtain a final concentration of 2.5* *μM DNA, 0.1 vol-% Triton-X100, and 0.15 wt-% CMC. Capture and control DNA sequences (detailed in the section “Results and Discussion”) were sprayed using the airbrush. The air pressure was set to 25* *psi, and the translation rate was 75* *mm min^−1^. Each line was airbrushed twice, allowed to dry for three minutes, and then airbrushed two more times. After airbrushing, the strips were allowed to dry in the ambient condition.

The paper strips were then blocked by airbrushing a light uniform coat of 1* *mg mL^−1^ BSA in deionized water and left to dry completely, then soaked in Tris-EDTA (TE) buffer at pH 8.0 with 0.3 wt-% BSA and 0.1 vol-% Tween-20. The paper strips were then rinsed four times in the TE buffer with 0.1 vol-% Tween-20. DNA hybridization was then carried out using either 250* *nM probe in 4× SSC buffer, 0.1 wt-% BSA, and 0.1 vol-% Triton X-100, or a mix of 250* *nM probe and 125* *nM target in the same buffer. The buffer without DNA or with SGC8 aptamer was used as a negative control. After hybridization, the paper strips were rinsed consecutively in 55 °C 2× SSC buffer containing 0.1% Tween-20, 2× SSC buffer, 1× SSC buffer, and 0.5× SSC buffer, then rinsed four times in Tris-buffered saline (TBS) buffer. These steps were designed to eliminate non-specific DNA binding.

To conduct chemiluminescent detection, the strips were placed in a solution containing 1:4000 dilution of streptavidin-alkaline phosphatase in TBS at pH 7.2, with 0.1 vol-% Tween-20 and 0.3 wt-% BSA. After being thoroughly washed with TBS containing 0.1 vol-% Tween-20 (TBST), the strips were exposed to 100* *μL of CDP-Star and allowed to sit for 20 min, then imaged using an Apogee KX32 camera (Apogee Imaging Systems, Roseville, CA, USA).

## Results and discussion

### Airbrushing condition

Before the demonstration of airbrushing small molecules, proteins, and DNA onto paper and glass, we studied a range of airbrushing parameters and their effects on line width. As mentioned above, airbrushes use the Venturi effect to draw a liquid from a reservoir to produce a fine mist of small droplets. Viscous instabilities occurring at the interface of the high-velocity/low-density air and low-velocity/high-density liquid are fundamentally responsible for the atomization of the spray. As a result, the feature size can be adjusted by controlling parameters related to the jet break up (for example, surface tension, jet velocity, and fluid density) and the areal deposition rate (for example, residence time and cone angle).

Several of these parameters are easier to adjust in a controlled and reproducible fashion, namely, solution viscosity, air pressure, airbrush translation rate, and tip-substrate separation distance. Among them, solution viscosity and air pressure primarily affect jet breakup, although they also affect areal deposition rate through variations in the flow rate. However, the airbrush translation rate and tip-substrate separation distance affect only the areal deposition rate. Using these key parameters, we are able to produce line widths ranging from ~500 μm to tens of millimeters, length scales comparable to consumer grade inkjet printing capabilities^19^. The line width was determined by using ImageJ (available from https://imagej.nih.gov/ij/) and averaged over multiple line segments.

**Figure 2:**
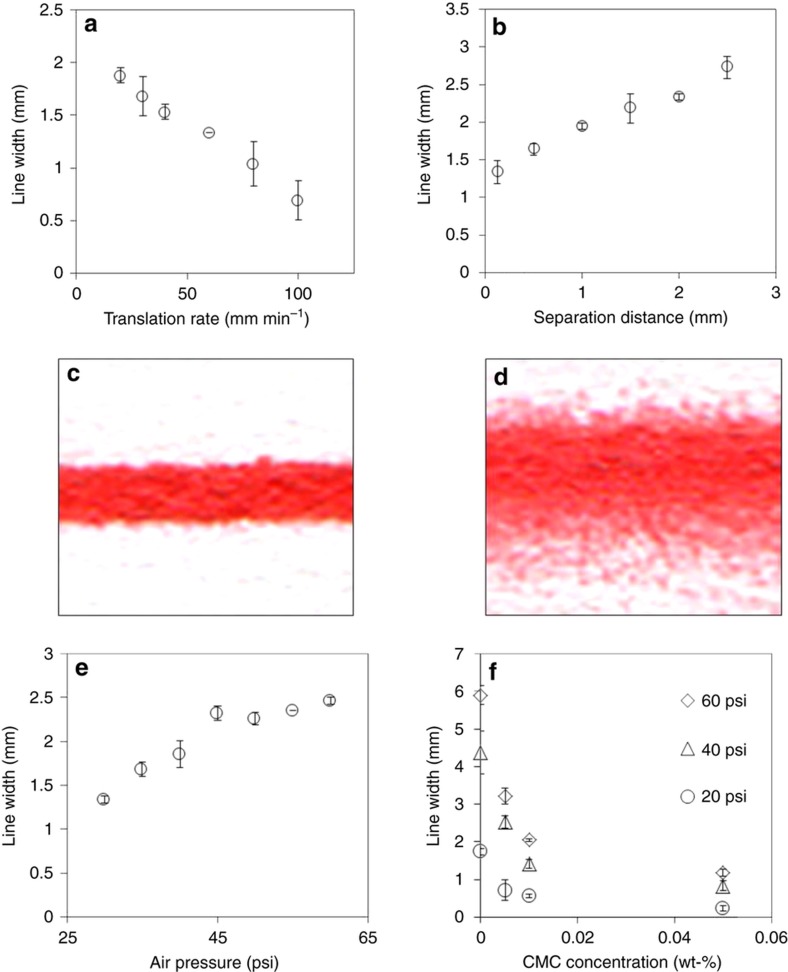
The effects of the airbrush operational parameters on the width of patterned lines. The parameters include (**a**) airbrush translation rate, (**b**) tip-substrate separation distance, (**e**) air pressure, and (**f**) CMC concentration at three different air pressures. Images of lines obtained with tip-substrate separation distances of (**c**) 0.125* *mm and (**d**) 2.5* *mm illustrate the smearing effect that accompanies increased line width. CMC, carboxymethyl cellulose.

The easiest parameters to adjust ad hoc are the airbrush translation rate and tip-substrate separation distance because they can be controlled directly by using a motorized translation stage. Both of these parameters affect areal deposition rate, and we found that they were linearly related to the line width. The airbrush translation rate (Figure 2a) had the largest effect for a given pressure, thus resulting in a line-width change of 2.7 times when the translation speed changed from 20 to 100* *mm s^−1^ (a factor of 5). The tip-substrate separation distance (Figure 2b) had a smaller effect on line width, changing only by a factor of two over a 20-time change in the separation distance (from 0.125 to 2.5* *mm). Because the spray from the airbrush is approximately conical, the increase in the separation distance results in a change in the line width, and some smearing of the line, as illustrated by the comparison of Figure 2c (at a separation distance of 0.125* *mm) and Figure 2d (at a separation distance of 2.5* *mm). Note that these results were obtained with an air pressure set at 30* *psi and the control screw at ½ turn after the needle valve was adjusted by closing it fully and then drawing it back by rotating the control screw.

The air pressure could also be controlled when the airbrush is set up. The effect of the air pressure on the line width is shown in Figure 2e. The trend can be explained by Euler’s equation along a streamline, $\frac{1}{\rho }\frac{\partial p}{\partial s}=-U\frac{\partial U}{\partial s}$, where *ρ* is the fluid density, *p* is the pressure, *s* is the axis along the streamline direction, and *U* is the fluid velocity^31^. The equation suggests that a higher air pressure increases the flow rate of the liquid through the nozzle, thus enhancing the rate of deposition.

We also tested the effects of the viscosity on the line width by adding a polymer, CMC, to the solution, which increased the solution viscosity. As shown in Figure 2f, the line width decreased with increasing CMC concentration. Viscosity has two effects on deposition. The first is related to the breakup of the jet and the atomization process, and the second is related to the decreased flow rate. Note that these results were obtained with an airbrush translation rate of 30* *mm min^−1^ and a separation distance between the airbrush and substrate of 0.125* *mm.

### Creation of lines and patterns

Airbrushes are superior to other non-contact printing methods for several reasons. First, unlike traditional inkjets and bubble jets, they have an infinitely variable orifice size, as defined by the position of the needle valve (Figure 1c). As a result, they can dispense a wide variety of viscous or particulate-based materials that tend to clog the nozzle of an inkjet. Second, the jet velocity is easily controlled through the coupling between pressure and velocity, thus making airbrushes less sensitive than inkjets and bubble jets to solution viscosity, density, and surface tension. Third, airbrushes are designed to use a wide variety of solvent-based paints; hence, even harsh solvents can be sprayed through an airbrush without causing any damage. Finally, airbrushes have a low acquisition cost, and waste little or no solution during operation, thus making them much less expensive to operate than inkjet printers.

One way to define the shape of the sprayed area with an airbrush is to rely on a mask, as we have demonstrated previously for the fabrication of microelectrodes^23^. Using a mask to control the spray pattern poses several challenges. First, a mask must be made before usage as in the screen-printing method. Second, overspray is desirable to cover the opening of the mask plus some of the outside of the opening, thus leading to waste. Third, the contact between the mask and the surface tends to have a space, which acts similarly to a capillary channel and allows the solution to wick to the outside of the boundaries defined by the mask. This wicking often leads to pattern distortion and loss of resolution.

**Figure 3:**
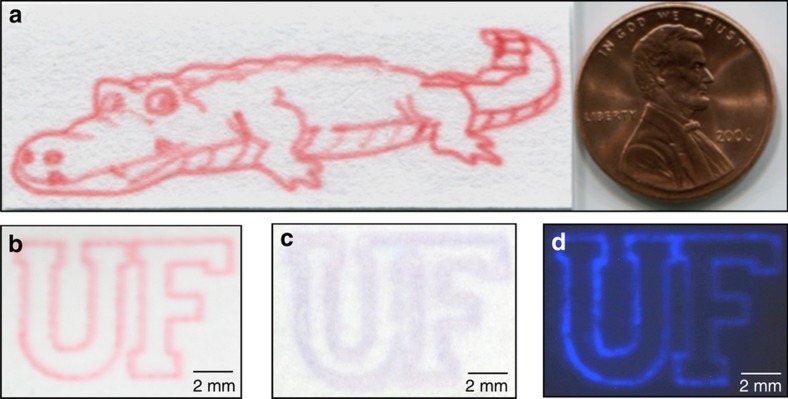
(**a**) An alligator printed using the airbrush. The drawing is composed of over 40 discrete lines, each with a width of ~500* *μm. It is shown to scale with respect to one US cent. (**b**) Red food dye airbrushed on paper in the shape of two letters U and F. (**c**) Colorless HRP was airbrushed on paper, and colorimetric detection was then performed using TMB. (**d**) Colorless HRP was airbrushed on paper in the shape of two letters U and F, and chemiluminescence detection was then performed with luminol and hydrogen peroxide. The image was captured using a long exposure camera. HRP, horseradish peroxidase, TMB, tetramethylbenzidine.

In this work, we demonstrated using an airbrush to pattern a variety of reagents, including dyes, proteins, DNA, and microparticle suspensions, in fine, reproducible lines or small diameter dots, without the use of a mask. Figure 3a shows a picture of an alligator made by patterning more than 40 discrete lines. These lines were formed by airbrushing a food dye solution onto a piece of paper while the location of each line was controlled by translating the airbrush in a predefined pattern. The start and end of each line were controlled by the trigger in Figure 1. The overall dimensions of the alligator are compared with those of a US penny in the same picture. The line width is ~500 μm, a value comparable to lines used in common lateral flow assays. The complexity and detail of the picture illustrate the flexibility, precision, and quality of airbrushing, all of which are similar to those of tattoos.

Figure 3b shows two letters, U and F, airbrushed onto a piece of paper with red food dye. We then replaced the food dye with a protein, HRP. Because the paper was white and HRP was colorless, there was no observable pattern after drying. After the paper was immersed in a solution of TMB, a colorimetric indicator, an image was observed as shown in Figure 3c. When a chemiluminescent indicator was used to replace the colorimetric dye, luminescence in the letter shape was captured on a camera as shown in Figure 3d. These results indicated that airbrushed proteins can be used to perform colorimetric assays and enzymatic reactions.

### Dot arrays by airbrushing

**Figure 4:**
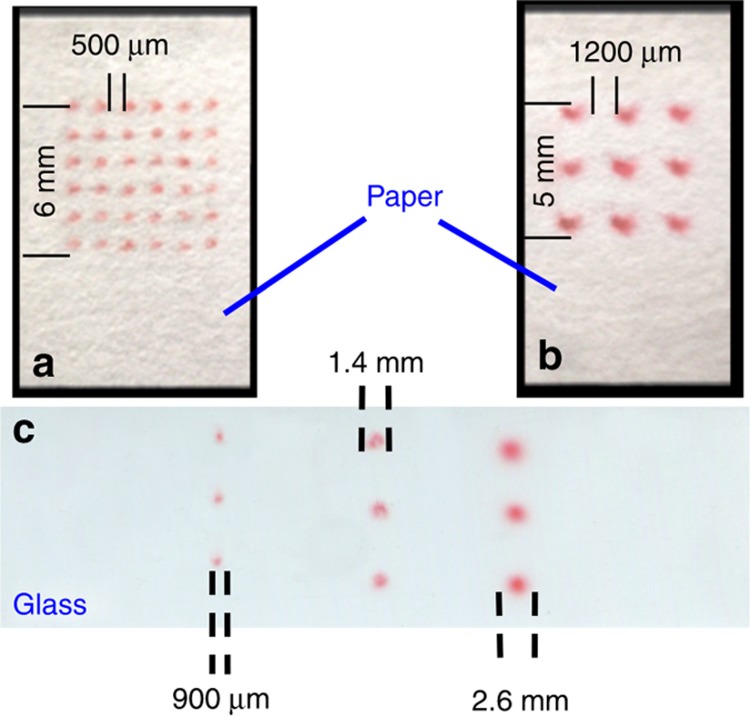
(**a**) A 6×6 array of 650* *μm dots on paper. (**b**) A 3×3 array of 1420* *μm dots on paper. (**c**) 1×3 Arrays of 900, 1400, and 2600* *μm dots on glass.

In addition to line patterns, we were also able to produce arrays of uniform dots of various sizes on paper, as shown in Figures 4a and b. Two sizes of dot arrays were patterned: a 6×6 array of 650* *μm dots and a 3×3 array of 1420* *μm dots. To produce the 650* *μm dots, the airbrush tip was held against the surface, and the needle valve was pulsed 8 times by pressing the trigger in Figure 1, leaving a one-second gap between pulses. To produce the 1420* *μm dots, the airbrush tip was held 10* *mm away from the surface and the needle valve was held open for 2* *s. Dot arrays are important in developing protein or DNA-array-based technologies.

The same method was successfully used to produce equally complex patterns on non-porous materials such as glass. Three sizes of dot arrays were patterned: (1) a 1×3 array of 900* *μm dots, (2) a 1×3 array of 1400* *μm dots, and (3) a 1×3 array of 2600* *μm dots, as shown in Figure 4c. A slightly different procedure was used to produce each of the three dot sizes. For the 900* *μm dots the airbrush tip was held against the surface, and the needle valve was pulsed 12 times, with a one-second gap between pulses. For the 1400* *μm dots the airbrush tip was held 10* *mm from the surface and the needle valve was pulsed 12 times, with a one-second gap between pulses. Finally, to produce the 2600* *μm dots, the airbrush tip was held 15* *mm from the surface, and the needle valve was held open for 2* *s. The dot size was determined by using ImageJ (available from https://imagej.nih.gov/ij/) and averaged over each type of dot fabricated.

### Conductive traces

**Figure 5:**
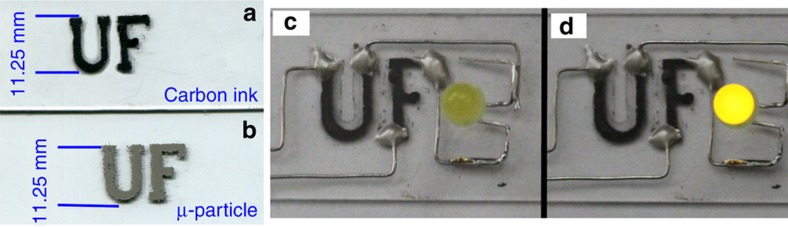
(**a**) Conductive carbon ink patterned on glass. (**b**) Metallic microparticle suspensions patterned on glass. (**c** and **d**) Conductive traces were connected with an LED when power was off (**c**) and on (**d**).

We also used the airbrush for patterning conductive carbon inks onto glass substrates. Airbrushed carbon traces may function as resistors and conductors for electronics, or electrodes for electrochemical detection^23,32,33^. Figure 5a shows an image of airbrushed carbon-based conductive traces. Similarly, we airbrushed the same pattern by using metallic microparticles (~6* *μm) Hiperco 50 powder, as shown in Figure 5b.

Conductive carbon paste was diluted with acetone to obtain a final concentration of 0.1* *g mL^–1^. Hiperco 50 powder was suspended in a solution of 0.1 wt-% Triton X-100 and 0.1875 wt-% CMC. Each solution was vortexed for 30* *s, and an aliquot of 250* *μL was pipetted into the reservoir of the airbrush. When the needle valve was opened one full turn, a uniform coat of carbon ink was deposited across the entire surface of the glass microscope slide. The carbon ink was allowed to dry for 5 min, while the airbrush was cleaned and loaded with pure acetone. The needle valve was closed until liquid just started to flow, and the pressure was decreased to 20* *psi. Carbon ink in areas around the pattern was removed by airbrushing acetone at the ink, using a milling machine to control the final pattern. After the unwanted carbon ink was removed, the glass slide was dried for two hours at 250* *°C.

To illustrate the conductivity of the airbrushed metal traces, we connected one end of each letter to a power source using conductive epoxy and the other end of each letter to a light-emitting diode (LED) as shown in Figure 5c. When the power was turned on, the LED lit up, as shown in Figure 5d, thus indicating the airbrushed metal traces were continuous. This finding was in agreement with previous results of microelectrodes and electronic contact pads made by airbrushing^23^.

### DNA hybridization

In addition to the protein assays shown in Figure 3, we conducted a paper-based DNA hybridization assay. We designed this assay for detecting influenza virus A, although it could be adapted to detect different types of viruses by changing DNA sequences and adding multiplexing capabilities. Influenza virus A is an RNA virus consisting of several genes that encode distinct proteins with various functions related to infection and replication. Two critical genes encode the two surface glycoproteins, hemagglutinin (HA) and neuraminidase (NA), which are the basis of the antigenic determination system used to classify influenza viruses (for example, H1N1)^34^.

**Figure 6:**
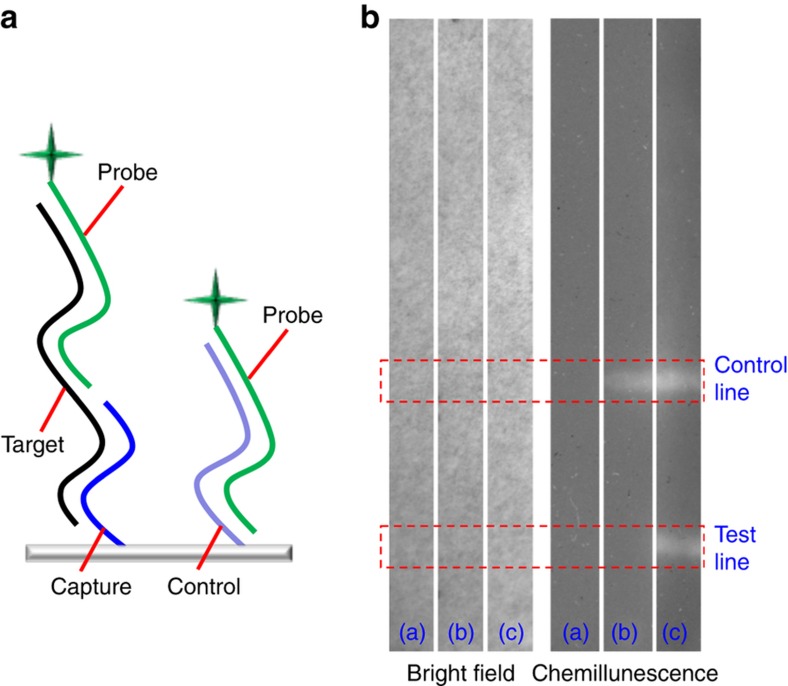
(**a**) Schematic illustration of oligonucleotide sequences. The capture and probe sequences are complementary to different parts of the target sequence. The control sequence is complementary to the probe sequence. Both the capture and control sequences are airbrushed onto the paper substrate. (**b**) Chemiluminescence DNA hybridization assay showing airbrushed control and test lines. The bright-field images on the left show no signals, and the chemiluminescence images on the right show the expected signals. Among three images in each condition, (**a**) The paper strip after treatment with buffer solution without DNA (that is, negative control), (**b**) The paper strip after hybridization with only the probe sequence, and (**c**) is the paper strip after hybridization with both target and probe sequences.

We chose a target sequence of the hemagglutinin (HA) gene on the basis of the results of Whiley *et al.*^35^ The target sequence was based on the combination of the forward and reverse primers as well as the target regions of the viral HA gene. The reaction scheme is shown in Figure 6a and the sequences of four nucleic acids used are listed in Table 1. The capture sequence is airbrushed onto the substrate and it is complementary to one-half of the target sequence. After the target is conjugated with the capture sequence on the substrate surface, the probe is then hybridized with the target, because its sequence is complementary to the other half of the target. The probe sequence is labeled with biotin, which interacts with streptavidin-alkaline-phosphatase for CDP-star-based chemiluminescent detection. As a positive control, a control sequence is immobilized in the control zone and its sequence is complementary to the probe, thus the control always yielded a positive signal unless the assay malfunctioned.

To carry out the DNA hybridization assay, the capture and control DNA sequences were airbrushed onto the paper surface by using the airbrush and were covalently coupled to the surface by using an approach based on chitosan and glutaraldehyde^29^. The test strips were hybridized with either a probe sequence or a combination of target and probe sequences. The results of this assay in Figure 6b showed that airbrushed DNA reagents can be used for detecting influenza viruses.

## Conclusion

We present a new method for patterning reagents on paper and other materials by using an airbrush. The flexibility and reliability of this method are shown by dot arrays and complex patterns on paper and glass by using a variety of reagents including small molecules, proteins, DNA, and conductive microparticles. This method is simpler, less expensive, and less wasteful than many other methods available, and it can make research in the areas of paper-based microfluidics more accessible. It is also possible to dispense hydrophobic reagents such as organic reagents.

Several limitations must be considered when using airbrushes to produce patterns. One limitation is the lower limit for the size of patterning features (for example, lines or dots), which is primarily defined by the needle valve diameter. However, the feature size in sub-mm should be useful in several applications such as paper-based microfluidic devices, electrochemical detectors^23^, and lateral flow assays, as demonstrated in this work. To function properly, the airbrush must be supplied with air long enough to draw solution out and then carry it to the working surface. As a result, the airbrush is more suitable to patterning lines/patterns than dots. Despite these limitations, we were able to produce a large number of line patterns on paper and array dots, and to perform protein assays and DNA hybridization in this work.

## Figures and Tables

**Table 1:** Sequences used for detecting H1N1 flu virus

Name	Sequence	Base Position
Capture	5′- ACCTTTCAAATGATGACACTGAGCTCAATTGCTCTCTTTTTTTTTTTT-3′–NH_2_	389–424
Probe	Biotin–5′- CATGATTGGGCCATGAACTTGTCTTGGGGAATATCTCAA-3′	426–463
Target	5′- GAGAGCAATTGAGCTCAGTGTCATCATTTGAAAGGTTTGAGATATTCCCCAAGACAAGTTCATGGCCCAATCATG-3′	389–463
Control	NH_2_–5′- TTTTTTTTTTTTGAGATATTCCCCAAGACAAGTTCATGGCCCAATCATG-3′	426–463
